# Salvage therapy with raltegravir in a 3-month-old infant

**DOI:** 10.1186/1758-2652-13-S4-P157

**Published:** 2010-11-08

**Authors:** AB Brolund, C Ilchmann, R Ganschow, O Degen

**Affiliations:** 1 University Medical Center Hamburg Eppendorf, Department of Paediatric Immunology, Hamburg, Germany; 2 University Medical Center Hamburg Eppendorf, Department of Medical Microbiology and Virology, Hamburg, Germany; 3 University Medical Center Hamburg Eppendorf, Ambulanzzentrum, Infections Diseases Unit, Hamburg, Germany

## Background

The integrase inhibitor raltegravir (RAL) is widely used in adults. Only limited data are available for children and no data for infants. We describe the case of a three-month-old infant treated with RAL in combination with lopinavir/r (LPV/r) and lamivudine (3TC).

## Methods

The mother emigrated from Ghana several years before and was insufficiently treated in a local hospital with AZT, 3TC and nevirapine (NVP) with constantly high plasma viral load (VL). At admission to the external birth clinic her VL was 160,000 copies/ml, the CD4 count 146/ml. The infant had a gestational age of 35 weeks with a birth weight of 1940g. A high-risk chemoprophylaxis with AZT, 3CT and NVP was given until the confirmation of a HIV1 infection three weeks later. The infant was then referred to our university hospital in an underweight state. His VL was 2.5Mio copies/ml, the CD4 count was 37% (2110c/ml). The genotypic resistance profile showed full resistance for all NRTIs and NNRTIs. We started an off-label therapy including RAL at 6mg/kg BID. The dosage was extrapolated from smaller trials in children ≥6 years of age. RAL is only available in 400mg tablets, so we pestled the tablet, attenuated the powder and distributed the required amount of mixture into a capsule. The content was then solved in water and administered by the mother. We combined RAL with LPV/r and 3CT BID which were dosed according to paediatric recommendations and adjusted monthly due to weight gain in closed cooperation with the pharmacologist.

## Results

The therapy was well tolerated, no clinical or laboratory adverse events have occurred yet. The boy showed a catch-up growth and weight gain from <3rd percentile to >25th percentile at week 16 of therapy. In the same period his VL decreased from 1.8Mio copies/ml to 164 copies/ml and his CD4 count increased to 39% (3357c/ml). We performed a PK-profile and measured sufficient drug levels of RAL and LPV/r, comparable to the limited data of PK-studies conducted in older children. RAL Cmin: 146ng/ml, Cmax: 1960ng/ml, Tmax: 2h.

## Conclusions

Due to the widely use of NNRTIs in developing countries an increasing number of mother-to-child transmissions of HIV with multi resistances can be expected in the near future. We describe a successful salvage therapy including RAL in a three-month-old infant. Further studies and investigation into the paediatric pharmacokinetics and application forms are warranted to confirm our results.

